# “We Got an Invite into the Fortress”: VA-Community Partnerships for Meeting Veterans’ Healthcare Needs

**DOI:** 10.3390/ijerph18168334

**Published:** 2021-08-06

**Authors:** Carol J. Ward, Curtis Child, Bret L. Hicken, S. Matthew Stearmer, Michael R. Cope, Scott R. Sanders, Jorden E. Jackson

**Affiliations:** 1 Department of Sociology, Brigham Young University, Provo, UT 84602, USA; cchild@byu.edu (C.C.); michaelrcope@byu.edu (M.R.C.); scott_sanders@byu.edu (S.R.S.); 2 VHA Office of Rural Health, Veterans Rural Health Resource Center—Salt Lake City, Salt Lake City, UT 84148, USA; Bret.Hicken@va.gov; 3 The Ohio Department of Medicaid, Upper Arlington, OH 43221, USA; Steven.Stearmer@medicaid.ohio.gov; 4 Department of Agricultural Economics, Sociology and Education, Penn State University, University Park, PA 16802, USA; jej5332@psu.edu

**Keywords:** community-engaged research, veterans, healthcare access, VA-community partnerships

## Abstract

Responding to identified needs for increased veterans’ access to healthcare, in 2010 the United States Department of Veterans Affairs (VA) launched the Veteran Community Partnership (VCP) initiative to “foster seamless access to, and transitions among, the full continuum of non-institutional extended care and support services in VA and the community”. This initiative represents an important effort by VA to promote collaboration with a broad range of community organizations as equal partners in the service of veteran needs. The purpose of the study is an initial assessment of the VCP program. Focus group interviews conducted in six sites in 2015 included 53 representatives of the local VA and community organizations involved with rural and urban VCPs across the US. Interview topics included the experiences and practices of VCP members, perceived benefits and challenges, and the characteristics and dynamics of rural and urban areas served by VCPs. Using a community-oriented conceptual framework, the analyses address VCP processes and preliminary outcomes, including VCP goals and activities, and VCP members’ perceptions of their efforts, benefits, challenges, and achievements. The results indicate largely positive perceptions of the VCP initiative and its early outcomes by both community and VA participants. Benefits and challenges vary by rural-urban community context and include resource limitations and the potential for VA dominance of other VCP partners. Although all VCPs identified significant benefits and challenges, time and resource constraints and local organizational dynamics varied by rural and urban context. Significant investments in VCPs will be required to increase their impacts.

## 1. Introduction

Healthcare systems for military veterans exist in many countries throughout the world and globally serve millions of veterans [1]. In addition, many countries make massive investments in veteran healthcare systems with annual budgets of nearly USD 4 billion in Canada, USD 11 billion in Australia, nearly USD 20 billion in the United Kingdom, and over USD 100 billion in the United States [2]. Additionally, veteran healthcare systems around the world typically combine medical corps and contracted services with civilian healthcare [1].

Examples abound. In the UK, for example, one initiative by the Armed Forces Veterans (AFV) involves integrating veterans into the national health services (NHS) [3], while other smaller initiatives and community-centered responses focus on improving veterans’ healthcare access as well as services to and relations with veterans [4,5,6]. Similarly, studies in Canada clarify the role of community organizations in assisting military families access civilian healthcare [7] and helping homeless veterans access healthcare and improve services for veterans [8].

These studies suggest that the complex health needs of vulnerable populations, including veterans, cannot be met by a single healthcare organization. The diverse social determinants that impact health require partnered strategies that share resources and expertise across multiple health stakeholders [9]. Of particular relevance to this study are healthcare partnerships focused on increasing the participation of interested community groups. Research on community-based interventions indicates that they have the potential for greater impacts on health-promotion and health conditions among vulnerable groups, such as low-income groups, persons with disabilities, and migrants [10]. In fact, recent studies focusing on health equity gaps indicate that community partnerships help to address the needs of historically marginalized or high-risk populations, such as people without homes, individuals within the criminal justice system, those with substance abuse disorders [11], minority language speakers, racial minorities, and LGBTQ persons [12]. Community-based partnerships depend on the active involvement of stakeholder groups in order to improve their effectiveness and sustainability, outcomes which are important but often complex and challenging to evaluate [13,14].

Indeed, collaborative, multi-sector efforts to address social and health challenges have become quite common [12,15,16,17], as reflected by the rich literature on the topic. Scholars point to several theoretical explanations for interorganizational relationships, ranging from resource dependence theories to transaction cost economics to institutional theories [18]. They examine not only the why, but also the how, of collaboration, including its benefits and drawbacks [19,20]. Of particular importance here, potential advantages include gaining access to resources (such as knowledge of beneficiary communities), gaining access to new markets, cost sharing, and organizational learning [18,21]. Disadvantages include the possibility of mission drift, loss of autonomy, and the time and resource costs associated with supporting collaborations [22].

Relationships between military and civilian partners can create uniquely complicated challenges to providing healthcare for veterans. Although previous research on the VA healthcare system has investigated policy and program changes within this system, there is a limited understanding of the crucial links between the veterans’ healthcare system and other community organizations that serve veterans [23,24]. Therefore, understanding how community healthcare partners’ perceptions and interactions shape access to veteran healthcare is essential. This study explores the US Department of Veterans Affairs (VA) system and, in particular, the Veteran Community Partnership (VCP) program, to more fully understand the new linkages among community stakeholders and the implications for veteran healthcare access.

In the US, 8 million of the 22 million veterans are enrolled in VA healthcare [25]. Importantly, about 25% of veterans reside in rural communities, and 57% of rural, compared to 37% of urban veterans, use VA healthcare. Additionally, 70% of older veterans use both VA and non-VA healthcare to meet their increasing needs as they age [25,26]. Without systematic caregiver coordination, the likelihood increases for discontinuous, redundant, or missed care [25]. To address the need for strong partnerships among VA and non-VA care providers, the VA developed the VCP Initiative to “foster seamless access to, and transitions among, the full continuum of non-institutional extended care and support services in VA and the community” [25]. Although VA provides much-needed healthcare to millions of veterans, studies have identified limits to its responsiveness to veteran needs and variations in access and care by rural-urban community context [27,28]. The VCP initiative aimed to address such concerns head-on. This study of VCP efforts to facilitate coordination of care through community healthcare collaboration provides valuable insight into ongoing efforts to improve veteran health. More broadly, the research allows community and public health scholars and practitioners an opportunity to consider the processes, benefits, and challenges of national-level bureaucratic efforts to build bridges among community-level healthcare providers.

VCPs are coalitions of veterans, caregivers, VA facilities, healthcare providers, and community organizations working together to support veterans and their families. The aim of the VCP initiative is to create local partnership networks that increase communication and collaborations among VA and their respective communities; improve coordination of care and services from VA and non-VA partners; increase opportunities for new enrollments; develop interpersonal and inter-professional contacts and relationships between VA and community organizations; and educate veterans, families, and healthcare providers on VA and community resources [25]. In the 2019 annual VCP report, VA reported VCP “sites in 61 VA Medical Centers (VAMCs) in all 18 Veterans Integrated Service Networks (VISNs) in 28 states” [25].

The standard VCP structure consists of a coalition of VA staff and representatives of community organizations that is co-led by a VA employee and a community member. Many sites also have formed a smaller steering committee of VCP members. As a community-driven model of collaboration, however, each VCP structure is individualized and has unique areas of focus, membership, and strategies for achieving its aims.

Relevant community-health focused research suggests that effective community health-promotion programs include structural interventions designed to increase community capacities, such as local leadership, partnerships, and resource mobilization [29]. This research suggests that organizational and social processes as well as the community contextual factors are crucial to understanding the effectiveness of community-based healthcare partnerships such as the VCP.

Using a multidimensional community framework, we focus on the experiences, practices, and relationships among local community partners as they pursue VCP goals [30]. Specifically, we examine the meaning and value community partners associate with the VCPs as well as the actions, practices, and interactions of VCP members [30]. Another important consideration concerns the geographic areas served, including structural and socio-spatial characteristics that contextualize VCP actions and dynamics. Thus, the purpose of this paper is to use a community-oriented conceptual framework to analyze VCP processes and preliminary outcomes, VCP goals and activities, and VCP members’ perceptions of their efforts, benefits, challenges, and achievements.

## 2. Materials and Methods

This report draws heavily from a 2015 pilot assessment that was part of a continuous quality improvement effort by the Office of Geriatrics and Extended Care and Office of Rural Health. A qualitative approach was selected that would address the research purposes of this case study. This approach included focus groups and interviews with VA employees and community volunteers who were involved with VCP initiatives [31]. To maximize the range of experiences that participants could address, all VCPs operating in 2015 were informed of this study during a regular technical assistance call, followed up with an email invitation. Given the research funds available, six interested VCPs, representing a geographically diverse set of communities and populations, were selected to participate in focus group interviews designed to address questions related to their experiences and perspectives on the VCPs and communities served by the VCPs.

To preserve VCP anonymity, details of locations and participants are not provided. The six sites were drawn from six geographically diverse Veterans Integrated Service Networks (VISNs) in 2014 that included both urban and rural areas. VCPs had approximately 1 to 5 years of experience, and three sites had existed previously as Hospice Veteran Partnerships (HVP).

Prior to the focus groups, one of the authors conducted a preliminary phone interview with the steering committee or the co-leads of each VCP. VCP leaders also completed a questionnaire addressing their leadership structure, membership, workgroups, and service area. The interview and background information guided the development of the focus group questions.

Focus groups, held between April and August 2015, were conducted at each site and facilitated by at least two researchers in all but one case. To increase the likelihood of candid feedback, informed consent was obtained and the research team assured participants that their comments would be confidential. Focus groups averaged 113 min, with an average of nine participants, for a total of 53 individuals representing 38 different organizations and agencies. Interviews were based on a common interview protocol but also utilized the background information received. Focus group interview questions asked for additional details on veterans’ health needs as well as VCP history, structure, priority goals, successes, challenges, and measures of outputs and outcomes. Audio-recordings and transcriptions of focus group interviews, and moderators’ notes and observations were included in the research database along with supplemental, follow-up information collected by email and phone after the focus groups concluded.

Analysis of the study data followed standard social science conventions for qualitative, case study research [32,33,34]. The research team used software, Dedoose, to code and analyze focus group transcriptions and other data. Using an iterative process, the researchers developed and used a coding guide to apply codes to text segments and, where necessary, revise or expand the coding guide to account for new insights. At least two researchers examined each transcript independently to increase confidence that coding was consistent and reflected important similarities and differences. Quotes selected represent patterns across all sites or that provide a telling insight into salient issues.

## 3. Results

In the following sections, we contextualize the VCPs across several dimensions and discuss goals identified by VCP participants. The analysis then focuses on these goals and discusses how VCP participants perceive their success in achieving them as well as the challenges VCPs continue to face.

### 3.1. VCP Structural and Spatial Characteristics

Because the VCP initiative itself was new, most VCPs included in this study were fairly new; some had previously existed as HVPs. The oldest VCP was established in 2011 and the youngest in 2014. Leaders of these VCPs typically included a VA employee and a community member who is often working in a hospice or medical-related field. On average, partnerships included 15 organizations, although this varied widely. VCP service areas also varied, with two operating in very urban and very rural areas, respectively, covering only one or two counties. Others covered multiple counties and cities, and one covered roughly half the state.

The VCP circumstances varied in other ways as well. For example, in some sites, VCP members had participated in other networks of service providers concerned with providing veterans and their families medical services and other types of care. However, the role of VA varied in these networks—in some, VA presence had been substantial, and in others it was either minor or absent altogether. Understanding the history and structure of each VCP helped elucidate VA’s current role in the partnership and some of the challenges VCPs encountered. For example, VCPs transitioning from HVPs already had a close working relationship with VA. Having a broader focus, these VCPs reported developing strategies for including other organizations and changing their identity in the service area. In contrast, for VCPs with histories of more diverse community-based networking in which VA played a less prominent role, the challenge was to develop new working relationships with VA.

Another dimension of the VCP context involved the number and density of services and resources for veterans in the service area. In some rural sites, for example, the scarcity of resources in the service area provided a significant impetus for VCP members to coordinate their efforts to address local veteran and family needs. Thus, including VA as a partner and improving coordination made a substantial contribution to increasing veteran awareness of and access to a range of programs and service providers. In contrast, in the larger urban areas, the number of service options for veterans in need was significantly greater, which presented a different context and value for the VCP. While the VCP, in this case, was also an important venue for improving information and coordination, these efforts typically included only a subset of the large number of service providers in the area. A final dimension of the community context was important as well: variation in the community presence of military and veterans’ organizations created different levels of recognition of veterans’ needs and support for greater outreach and improved service coordination.

### 3.2. VCP Goals and Practices

**Figure 1:**
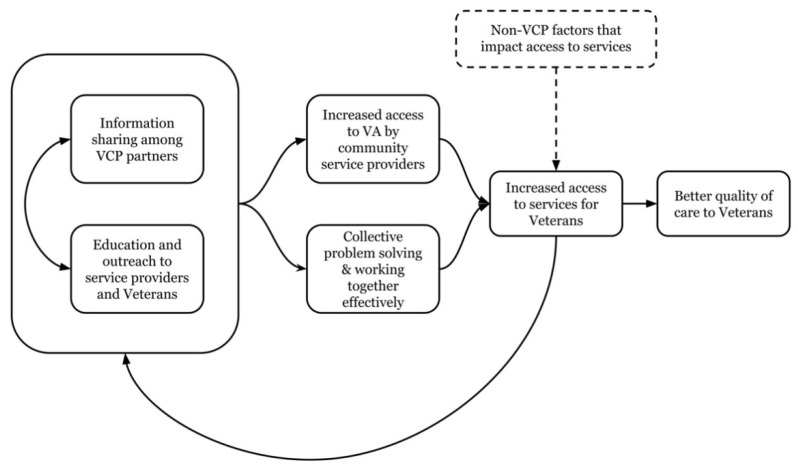
Relationships among VCP goals identified by focus groups.

Although there was some variation in the VCP goals and activities, goals addressed six overlapping areas: (1) increase of information sharing and collaboration among the partner organizations; (2) increase of the VCP’s educational efforts about VA and the partner organizations that serve veterans in their communities; (3) increase of access to VA programs and services for community partner organizations; (4) engagement in effective problem-solving related to veterans’ needs; (5) working together effectively to accomplish goals and activities; (6) the increase of services to veterans. Each goal represents a dimension of VCP efforts to successfully achieve the ultimate aim—improve Veteran access. These are represented as a multidimensional process in Figure 1. Importantly, information about VCP goals indicates the stage of development of the VCPs, specifically, their emphasis on developing the capacities of the partnership itself through mutual education and information sharing, developing common goals, solving problems, and working together effectively. Goals 1 through 5 are the focus of this paper not only because they were identified as priorities by VCPs from various contexts, but also because increasing information sharing and education as well as access to VA services, collaboration and collective problem solving were realistic pursuits given the resources typically available.

Figure 1 shows how the VCP members’ goals can work together to provide better quality care to veterans. As the figure illustrates, information sharing among VCP partners influences and is influenced by the VCP’s education and outreach efforts. Together, these increase community service providers’ access to VA and foster the VCP’s capacity to solve problems and work together effectively. Addressing these goals promotes increased veteran access to and use of services. Of course, access to services is also shaped by factors other than the work of the VCP, represented by the box with the dashed line and arrow. For instance, whether the VCP is located in a primarily urban or rural setting, the military presence in a community, the nature and availability of VA and non-VA services, family support, and access to transportation shape veterans’ access to services. Finally, as indicated by the return arrow, increased access to services provides feedback for the VCP’s current activities and shapes future efforts.

### 3.3. Success in Achieving VCP Goals

Most participants in the six VCP focus groups expressed positive opinions about the VCPs, indicating that it is a useful way to connect with VA and other community organizations. The following sections present focus group results for goals 1 through 5 (listed above) and include focus group quotes illustrating central themes and patterns in as well as variations in views among the VCP focus groups.

#### 3.3.1. Goal: Increase Information Sharing and Collaboration among the Partner Organizations and Agencies

This goal focuses on information sharing among VCP partners and with other service providers. Thus, the pursuit of this goal supports successful pursuit of the remaining goals.

Several of the VCP focus groups, particularly those whose members had prior experience in community-based networks, discussed their need for more information about VA services and for contacts at VA. This was a major problem that predated the VCPs and an important reason for joining the VCP. The formal and informal information sharing that occurs with VCP helps to decrease the “mystery” about VA, a benefit reported in every focus group. Conversely, some VCPs reported reciprocal information sharing as VA learned about community services that may benefit enrolled Veterans, which occurred as VA representatives coordinated with other VCP partners. However, since increasing access to VA is a major goal for most of the VCPs, the flow of information from the community to VA was discussed somewhat less.

For most of the VCPs, sharing information began with the VCP partners educating the other partners about the services their organization offers to Veterans and their families. This information is then used by the VCP partners as they work with Veterans to meet their service needs. The information sharing process described by a VCP member in one site is similar to the information sharing reported by most of the VCPs interviewed:

“*At the beginning of the meeting, participants would give a presentation about what kinds of services they provided. That was more beneficial because even though I might know what [Organization name] is, I don’t know … I don’t work for [Organization name] so I don’t know what services, all the services they provide or what’s available to Veterans. So, it was nice hearing that information and to be able to take that information back to the Veterans that we have or their families or caregivers or some people that are just walking in for information*”.

The information sharing that occurs as part of VCP participation often helped overcome the gap in knowledge about VA programs and services. Some VCP partners viewed this gap as a consequence of VA’s relative isolation from the community prior to their involvement in the VCP. One VCP focus group member describes the benefit of overcoming this information gap about VA services:

“*Okay, VA is in their box, so to speak and they know what programs they have. We’re in the community caring for Veterans and we have many different Veteran resource programs within the community. We don’t really know everything that VA can offer those Veterans. VA doesn’t really know what’s in the community outside of their box so it was a great place for us to understand … [The VCP] became the liaison between two sides; between the community and VA. That’s what we’ve been doing basically*”.

Importantly, some VCP members reported that, despite the fact that they had served Veterans prior to joining the VCP, the new partnership improved their ability to help Veterans because they could now share information about other community and VA resources. One VCP member’s comments echo those voiced in several VCP focus groups: “Personally for me it’s helped out … [P]rior to the VCP I really didn’t know what other resources were out there and what other organizations that VA contracted with, to line up services for the Veterans I serve”. Participants appreciated that as their understanding of each organization expanded, they developed a network of VA and community contact points who could assist with specific Veteran needs.

The information exchange that occurs as part of each VCP also increases feelings of mutual trust and good will and makes it possible for partners to collaborate on common goals. As one VCP member commented, “That has been helpful just having the resource of people sitting around these tables, knowing that I can call them and knowing that they have the same common goal [to serve] our Veterans and work with VA”. An important aspect of the information sharing identified by several VCP members is the fact that VCP members put the goals of the VCP ahead of their own interests, as expressed in the following comment:

“*And the nice thing about the hospices in our group is we talk … we do no advertising. This is strictly working together and if this group comes up with something that works well, the nice presentation they did, they share it with the group and the group can use it, so they like that*”.

All of the VCPs reported the benefits of sharing information. In fact, this activity was central to the development of each VCP’s efforts to develop an effective VA-community partnership. Of course, the success of information sharing among VCP members, to some extent, is affected by the continuity of interactions among VCP partners. When VCP members attend meetings infrequently or leave the VCP, the effectiveness of information sharing activities for coordination of events and services to Veterans may be attenuated. The challenges VCP members identified in relation to turnover and attrition are discussed in the next section.

#### 3.3.2. Goal: Increase the VCP’s Educational Efforts about VA and the Partner Organizations That Serve Veterans in Their Communities

This goal encompasses formal and informal outreach and educational activities that disseminate information about Veterans services to other community providers, Veterans, and caregivers. Education and outreach to providers and Veterans support all other VCP goals related to improving Veterans’ access to healthcare and other services.

VCP partners typically view their education and outreach efforts as a substantial step forward in improving services to Veterans. Participants almost universally agreed that VCP has promoted greater understanding among the partners about both VA and community organizations. Of particular importance, because of the coordination that occurs as part of VCP participation, VCP partners are becoming more aware of other Veteran-focused partners and activities occurring in their communities (e.g., stand-downs, information events, etc.). One VCP member’s comment shows appreciation for the unique circumstances and opportunities that the VCP provides. This VCP partner focused on the greater understanding that had been achieved among the partners, including VA, and saw the VCP activities as a pioneering effort as well as a challenge:

“*I think we’re at a unique point in history right now where, you know, we’re developing these partnerships and better understanding on both sides, VA and the communities, and it’s really exciting to be a pioneer on that level, very challenging, but there’s still so many unknowns, that neither side aren’t even aware that they don’t understand*”.

Additionally, VA’s new role as a partner means that perceptions about their role as the primary organization that serves Veterans are beginning to change. The following quote illustrates how one VCP member, a VA representative, expressed this change in perception:

“*I think it could really benefit the way that we network and stop seeing ourselves as Big Daddy, and just see ourselves as a player. Because the majority of our Veterans will actually be served by the community. They won’t be served by VA*”.

Efforts to increase awareness among the community organizations also include increasing awareness within the organizations that the VCP members represent. When VCP members share information obtained through the VCP with other staff and supervisors in their respective organizations, the awareness of and appreciation for Veterans’ issues are more broadly disseminated beyond the borders of the formal VCP membership, as illustrated by one VCP member’s comments:

“*Our company is large and we have so many various entities in it that whatever I gather here I took it back to the leads of the departments, that whatever we spoke about here that would pertain to their departments I felt responsible to share that information. And, also, if I knew something was on the agenda upcoming, to invite them to come and listen because then it’s a better service*”.

This increased awareness helps to strengthen and enlarge the network of Veteran advocates in a community. In several VCP focus groups, participants also discussed how their efforts in relation to the outreach and education goal have contributed to increased awareness of local services for Veterans among organizations and community members. For example, one focus group member described increased awareness among community members: “People in the community are starting to actually see at some level, what there is. It’s just a little bit more awareness of what each group does”. A member of a different VCP focus group also commented on feedback received from the community suggesting the success of VCP outreach and education events: “We’ve done enough events now that the community and more and more partners at VA Medical Center are, you know, they’re thinking, ‘Well, when’s the next event? What’s it going to be?’ And you know, we keep reaching out and asking, you know, what do you want to hear about? What do you want to learn about? So, I think we’ve done a good job of that”.

In another VCP focus group, a community representative was particularly positive about the ability of the VCP to successfully organize informative community events:

“*So, my biggest benefit is that we come here and we discuss and things happen (snaps fingers), you know? Events are happening, and it’s like wow! There’s actually motion, moving forward …. It’s not just everyone comes around once a month and talks and nothing comes from it*”.

VCP partners typically reported substantial benefits from their educational efforts. Not only did they perceive improved awareness among local organizations and service providers, but new levels of awareness among community members. This includes better understanding of some critical needs and challenges Veterans face and new awareness of the resources available to Veterans and their caregivers. Educational activities require little to no funding to organize and are less time demanding, so they fit more easily within the scope what VCPs feel they can accomplish with limited resources. Importantly, the benefit of pursuing this goal was expressed by all VCPs, regardless of the history of the VCP and whether the service area was large or small, urban or rural.

#### 3.3.3. Goal: Increase Access to VA Programs and Services for Community Partner Organizations

A third VCP goal differs from the previous two in its focus on VCP partners developing or extending working relationships with VA programs and services. This goal emphasizes the increased access to VA that community organizations gain through VA contacts they meet in the VCP network. These contacts are central to improving Veterans’ and caregivers’ access to VA programs and services.

As suggested in the previous section, many VCP members feel (or have felt) that VA is a “black box” that is difficult to understand and navigate. A recurring theme across all the VCP sites involved in the study is that the VCP has given VA a human face, making it more accessible. The VCP provides its members with a chance to have a different type of access to VA, as suggested by the following quote from a member of a recently organized VCP:

“*It was like we want to arrange a meeting between mental health providers in the community and the folks here and because I was on VCP they called me and said get some of your folks together. So I was like, ‘Hey, you know what? We got an invite into the fortress!’ So, I got some of my cohorts out here real fast and it was the initial meeting. And it was just like this is who we are, who are you? … But it really was, when I made the phone calls, everyone was like, ‘Are you kidding? They want us to come?’”*

It is significant that the act of inviting other community members to collaborate was both remarkable to the community partner, whose response affirms that such invitations were unexpected, and readily received. VCP members now know people they can call for questions and concerns. Access to VA has improved because the VCP has facilitated a more efficient avenue for coordinating with people and resources at VA. When asked to make a comparison of the current situation with how things were working prior to the VCP, one VCP member commented, “I’ve actually been in my position four, five years and with VA for 8, and it did not happen like that before”. She went on to explain, “It was more just conversation with the case manager at the hospital, and they would contact VA or they would do that and it took a lot longer; it was a longer process. However, now that I have the contacts and the direct connections, it’s a lot quicker process. It gets the Veteran the services they need quicker”.

Another VCP member’s comments focus on the benefits of the communication afforded by the VCP: “And what’s different about VCP is that it’s a conversation. … it’s a discussion across the board, so it allows the options to kind of be available … you know, the community says, “Hey, we do these things,” and VA says, “Hey, we do these things”.

Members from each of the VCP focus groups expressed agreement with the value of having knowledgeable contacts in VA, as suggested by this comment: “Now I know, [VA employee name] is a wealth of knowledge, a great resource when it comes to certain pieces of VA”.

In essence, increased access to VA through the VCP means that VA is no longer an isolated entity in the community: “VA was kind of a separate entity, and so this was kind of a way for us to kind of get together and network and, you know, educate each other on what we do and how we access those services”. Additionally, having VA as an equal partner in the VCP is a new experience for several of the VCPs, especially in more rural areas. They not only appreciate VA partner(s) as a way to get to know more about the resources and programs of VA, but also the opportunity to develop a new working relationship or expand prior networking. Indeed, in rural areas, the value of the VCP model was especially apparent because the prior VA participation with local provider networks had been less substantial than in more dense urban areas.

Accessing VA and relating to VA in new ways is a central goal of the VCPs interviewed. All the VCPs identified benefits related to their efforts on this goal. The VCPs who expressed the most benefits, however, were those whose professional networks had not previously involved VA. Consequently, the impact of having access to VA was perceived as a substantial gain.

#### 3.3.4. Goal: Engage in Effective Problem-Solving Related to Veterans’ Needs

An important VCP goal mentioned by all focus groups is effective problem-solving, which concerns the capacity of VCPs to address specific local needs and problems related to improving services to veterans. According to focus group participants, the VCP provides a useful forum in which community members and VA representatives discuss veteran needs and make plans to address them.

Importantly, collaboration was the hallmark of most VCPs. The following quote from one focus group discussion illustrates the collaboration of VCP members as they discussed solutions to specific needs—in this case, the need for a caregiver support program for recent veterans:

“*[Veterans] can reach us directly. However, there just has to be a bridge. And that bridge has to be well known. Otherwise the bridge is no good. … You need an artery of communication that’s known on both sides. And they have to have somebody they can reach that says, “You know what? That’s an excellent question. I just don’t happen to have that. Give me 24 h and I’ll find out”. That’s what they need to hear. You know, because they’re going to be empowered*”.

In another focus group, a VCP partner shared information about efforts to build relationships with agencies outside the VCP that could help provide information to the public about services available in a large service area.

“*I started hitting up the communities, the chamber of commerce. I started hitting up the commissioners for each of these cities … saying, ‘Listen, this is what we’re doing, and without your help. Now, what can you do for us to help put us in front of the community people that we need to be in front of?’”*

The benefit of collective problem-solving was very evident during some focus groups as the discussion would raise questions about a particular veteran issue, and VCP members almost automatically began to engage in the emerging practice of problem-solving which is central to VCPs. VCP focus groups highlighted this ability to collaboratively generate solutions to veteran problems as a VCP strength. One focus group participant’s comment illustrates typical views of the VCP’s successful efforts, “I think this group does a very good job at looking at the obstacles and trying to overcome them”.

VCP success in addressing local needs and problems varied in relation to the VCP community and its history. VCPs serving more rural areas faced greater challenges in finding resources to address identified needs. On the other hand, rural VCPs had close working relationships that provided a supportive context for their efforts. One rural VCP member described the benefits of rural location: “And maybe that’s easier done in a rural community because there’s not 1000 of us [local organizations] out there, there’s maybe a core dozen of us”. In contrast, while VCPs in large urban areas typically had more local or regional resources to recruit for solving problems, they also faced other challenges, such as the scale of the needs identified in their service population and the rate of turnover among the VCP members. Attrition and turnover among VCP members affect the abilities of the VCPs to work together effectively.

#### 3.3.5. Goal: Work Effectively to Accomplish Goals and Activities

The importance and benefits of having a durable working relationship and opportunity to coordinate care for veterans are reflected in a VCP member’s comment that, “… this is not a short term, one-time seminar. This is an ongoing relationship where we grow together, and that’s a different view”. In fact, a member of a different VCP in which there were strong relationships among VCP members, commented, “I mean, we know each other so … It’s almost like we work for the same boss and just our paycheck comes from somewhere else”.

Discussing the benefits of coordinating community events, a VCP partner in one rural site described a successful community event: “But the good news is … those veterans who attended [the] VCP [event] that we had two weeks ago to get information, to get connected. So that’s, I think a good example of how the VCP can operate to make things better, to reach out”.

Collectively organizing VCP-sponsored events also provided members an opportunity to represent the VCP, which suggests pride in VCP efforts to coordinate community outreach as well as VCP membership.

“*At least seven of my competitors sit on the partnership and when we do an event in [local town], we do an event together. …instead of my nametag from [my organization], I will wear a Veteran Community Partnership—either a baseball cap or a polo shirt—so we’re representing the partnership …. And we found that it’s working out nicely*”.

Focus group members also noted that VCP membership confers legitimacy on efforts to increase access to VA and generally improve services to veterans in the community. It was easier for community partners to justify the time spent working with other community members if they could do so under the auspices of an official government-supported initiative such as the VCP. Additionally, some VCP members believed their efforts were better organized through the VCP.

“*As a result, the services for Vets within the community here … it’s better organized and that it will serve the Vets in a better way, and the VCP has then helped. It’s just a gift. Put some emphasis behind it or legitimize it*”.

Some VCP community partners reported that their supervisors provided more support because of the official link to a federal initiative carried out at the local level.

“*My employer, actually they see this as a positive. So, it’s on the job description, “supposed to have a presence in the community”. So, they actually love the fact that I’m working with VA*”.

### 3.4. Challenges to Meeting VCP Goals

Notwithstanding the many benefits that focus group members reported, VCPs faced a number of challenges to achieving their potential. Many of the challenges relate especially to VCP members working together effectively.

#### 3.4.1. Material Support for the VCP

Some VCPs struggled to operate without a dedicated budget. Or, at least, they felt that they could do more with more resources. Given their budget constraints, increased information sharing and collaboration for problem-solving were the easiest and most sustainable activities for most VCPs. Even this activity could be difficult, however, because VA representatives often were not empowered to do much more. For instance, VCP members wanting to publicize an event as co-sponsored by the VA typically had to work through VA’s review and approval process, even though a VA representative was in the VCP. Such reviews could be difficult to obtain and time consuming, thus frustrating VA representatives on the VCP as well as other members not accustomed to working through a large bureaucratic system.

With a budget, for example, some VCPs reported they might create a website and post videos of speakers or links to resources. However, even when the VCP did have access to funds, managing those funds created additional workload and stress. One VCP site had received a small amount of VA funding, which was administered by the local Community Based Outpatient Clinic (CBOC). None of the VA members of the VCP or the CBOC staff had experience managing VA funds and were unprepared for the administrative challenges in obtaining permissions, locating vendors, or accounting for expenses. VCP members at this site suggested that the amount of work created to manage the funds somewhat obviated the benefits.

The lack of resources in VCPs may disproportionately impact rural areas. “If we’re trying to maximize our resources, which are very limited,” one interviewee observed, “it’s hard to reach those rural populations because it’s less bang for your proverbial buck”.

#### 3.4.2. The Role of VA

Although the collaborative aspect of the VCP venue was typically appreciated, at some sites VA had become the dominant leader of the VCP. In such cases, community partners looked to VA, rather than to the VCP, to make decisions or to act on issues that needed to be addressed. As a result, the VCP reproduced the hierarchy that it was intended to challenge, and the VA ended up shouldering the blame for VCP shortcomings. Likewise, VA staff who co-chaired these types of VCPs felt overworked and, ultimately, that the success or failure of the VCP rested on their shoulders.

In fact, some VCP members said that the partnership was successful precisely because the VA did not assume a strong leadership role. “I think it took a few months to realize,” one interviewee said, “it wasn’t like VA kind of running the show; it was kind of all of us together running the show”. The VCP members concluded that, “The more we do these things as a community, the less it will feel like VA is taking over because it really isn’t about VA. It’s about the community and serving our veterans”.

A delicate balance is required, therefore, for a VCP to flourish. While it seems imperative that VCPs include able representation from the local VAs, focus group data suggest this should always be balanced with leadership from the community.

#### 3.4.3. VCP History and Community Networks

VCPs that developed in contexts with more diverse community-based networks had a different range of experiences than VCPs with fewer networks. In some cases, VCPs faced challenges related to competing with other networks in the area or attracting organizations that could contribute to the VCP’s efforts. These challenges tended to vary by urban and rural context. Members of VCPs serving largely rural areas typically knew the local organizations well and had relationships that benefited VCP coordination efforts. Several VCPs located in larger, more urbanized areas also used their prior community networks to attract partners. However, these VCPs also competed with other veteran networks serving portions of the very large VCP areas. Among the challenges they faced were finding ways to diversify their VCP membership and attract difficult to reach groups.

Likewise, participants commented on the difficulties of attracting new community partners (e.g., veterans’ groups and service providers) because of their involvement in other service provider networks. For example, one VCP partner commented: “I’ve really been trying to get the service organization … and it’s not that they’re negative or anything, I mean, it’s just been kind of hard to… get them interested in kind of participating”. In some areas, VCP member involvement with another network also affected their ability to engage fully in the VCP. For example, VCPs that had transitioned from HVPs to VCPs noted that their continued involvement with national hospice and end-of-life care associations sometimes reduced their time for the VCP.

## 4. Discussion

What happens when a complex bureaucracy such as the VA makes efforts to build bridges in communities to collaboratively improve veteran healthcare? Data collected for this study of the VCP program from focus groups in six diverse sites, including VA and community partner organizations, suggest that focus group participants largely viewed the VCP initiative as a success. The significance of developing and sustaining these coalitions with little financial or administrative support, given the limited history of VA/community collaboration, should not be underestimated. Generally, VCPs have led to improved relationships, trust, and collaborations between communities and VA.

Regarding the VCP goals discussed, focus group analyses indicate that VCPs have facilitated organization-level outcomes, such as information sharing, communication, and effective problem-solving that address veteran needs in their service areas. These are some of the benefits of collaboration identified in the literature [18,21]. However, organization-level processes and outcomes vary by VCP, indicating the diverse conditions and challenges that community-based organizations face in creating effective collaborations.

Some VCP sites also reported veteran-level outcomes—improved care coordination and increased access—though these outcomes were primarily evident among VCPs that had been HVPs. Because the VCP initiative supported adapting practices and coalitions to local conditions, VCP necessarily works differently at every site. Although this approach allows partnerships to be responsive to local needs and changes, it also hinders the development of a common set of metrics for VA assessment of outcomes and VCP effectiveness across all sites. Typically, flexibility is seen as a benefit to interorganizational relationships [18]. In this case, however, flexibility itself has a cost. However, consistent with the literature, the collaborative relationship did impose on organizational autonomy. While VCP participants acknowledged the importance of such assessments, they also reported concerns that measurement efforts would place additional demands on their already stretched resources and detract from VCP priorities.

To address its mandate to assess VCP effectiveness, in 2017, VA created a VCP Evaluation Taskforce “to develop a set of metrics to pilot with a small sample of VCPs” [35]. Importantly, data from the pilot VCP evaluation sites indicated results similar to those reported in this paper. The most frequently cited benefits include “… developing/strengthening relationships and improving communication between VA and community organizations and agencies … increased VA’s involvement with community activities, and vice versa, and … continuity of care to meet the needs of veterans and caregivers” [35] (p. 14). VCP coordinators also reported challenges similar to those presented in this paper [35] (p. 15). Subsequently, the 2017 pilot evaluation led to the development of an online VCP Reporting System. The data presented in the 2018 VCP Summary report as well as the 2019 VCP Annual Report indicate that VCP activities, benefits, and challenges remain very similar to those identified in both this paper and the 2017 VCP Summary report [36] (pp. 14–15). The continuity of the VCP annual report findings suggests the value of the data presented here: as the older VCPs continue and new VCPs emerge, collectively they develop effective communication and working relationships among community partners and implement activities that benefit veterans and their families. However, this growing group of VCPs also face persistent challenges that represent potential threats to their effectiveness.

These findings are consistent with data from international studies of partnered healthcare interventions. For example, a 2017 analysis of three projects to introduce accountable care in Nepal, the Netherlands, and Germany identified the importance of creating organizational structures that foster trust among key stakeholders and encourage accountability [37]. In a 2017 study by Estacio et al., a local public health department and a local university in a UK community partnered to improve community health literacy over a three-year period [38]. The authors identified the importance of shared vision and goals for maintaining enthusiasm, direction, and momentum in partnered activities. The authors also recognized that synergies occur with diverse partners who contribute unique knowledge, skills, and experiences to problem solving. Recognizing the value of diversity engendered a spirit of trust and equality among the partners. Moreover, mutual learning, networking, and open communication were also described as essential components to a successful partnership. In Australia, an analysis of a community health program for youth reported that while trust is critical for partnership, trust alone will not produce successful outcomes unless underlying cultural factors that work against the partnership goals are addressed. Partnerships must consider strategies to address culture change and conflict if they are to be sustained in the long-term [39]. Finally, A 2004 study of community health nurses engaged in multiple collaborative projects in South Africa reported similar perspectives—the importance of goal-alignment and anticipating that conflict will eventually emerge. They also noted the importance of early participation as an “investment” as the benefits of participation may not accrue until the partnership matures [40].

## 5. Conclusions

The question remaining is how best to implement this VCP model. The successes reported here underscore the need to understand key elements of this community-level structural intervention that contribute to their effectiveness: the meanings associated with VA-community partnerships, their practices, and the power dynamics and relationships among partners in each rural and urban context [29,30]. Importantly, although many VCPs are led by VA representatives with a passion for this work, reliance on VA representatives makes the VCP vulnerable to such factors as staff burnout and turnover. Additionally, despite substantial benefits, VCPs struggle with other threats to their sustainability and scope, such as competing interests and networks, and insufficient material and administrative support. Addressing threats to continuing stakeholder engagement will require greater investment by VA and community partners in the VCPs. A limitation of this study is its focus on the VCP model in the US. However, increased community investments and partnerships are likely universally needed in veteran healthcare systems. Thus, comparative research is needed on community partnerships addressing veteran health needs in other countries. Future research should examine, for example, the effectiveness of community-based partnerships in other contexts, and the contextual and other factors that support effective solutions to veteran healthcare needs and challenges.

## Data Availability

The datasets generated during and/or analyzed during the current study are available from the corresponding author on reasonable request. The data are not publicly available due to the inclusion of information that could compromise the privacy of the research participants.
